# Stable polydisperse free-standing porous films made by mechanical deformation†

**DOI:** 10.1039/d4sm00569d

**Published:** 2024-08-02

**Authors:** Hsiao-Ping Hsu, Kurt Kremer

**Affiliations:** a Max-Planck-Institut für Polymerforschung Ackermannweg 10 Mainz 55128 Germany hsu@mpip-mainz.mpg.de kremer@mpip-mainz.mpg.de

## Abstract

Using molecular dynamics simulations, we show that the methodology of making thin stable nanoporous monodisperse films by biaxial mechanical expansion and subsequent cooling into the glassy state, also works for polydisperse films. To test this, a bidisperse polymer system of an equal number of very long (≈72 entanglements) and short (≤4 entanglements) chains with a polydispersity index of 1.80 is considered. The void formation and the development of the local morphology upon expansion, relaxation, and cooling are investigated. As for the monodisperse case, long chains in thin porous polydisperse films extend over several pores, stabilizing the whole morphology. The short chains do not fill up the pores but tend to aggregate inside the polymer matrix and to avoid surface areas and reduce conformational constraints imposed by the surrounding, a scenario very similar to strain-induced segregation between the strained long and relaxed short chains.

## Abstract 2

Nanoporous polymer film made by biaxial expansion of a polydisperse polymer melt. The film is stabilized by long polymer chains extending over several pores. Short chains aggregate inside the bridges between pores.

## Graphical abstract

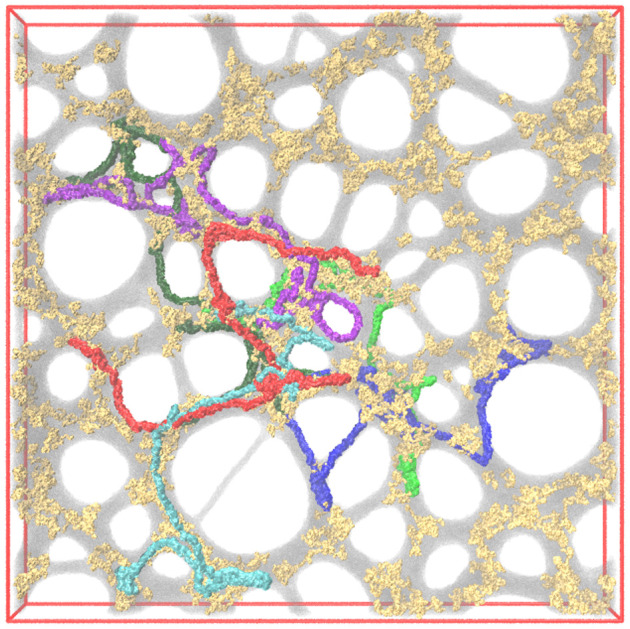

## I. Introduction

Porous polymer membranes are of high scientific and technological interest because of their potential applications in many fields such as water filtration, fuel cells or in pharmaceutical and biotechnology industries. This wide field of potential applications makes robust and simple preparation procedures very desirable.^1,2^ Typically the preparation of (nano)porous films takes advantage of incompatibility of components. Thus, a variety of block copolymers^3^ have been employed for designing nanostructured porous materials by changing block volume fractions, molecular weights, thermodynamic interactions between chemically different blocks (incompatibility), and solvent qualities.^4–9^ A collection of recently developed synthesis methods of generating micro-, meso-, and macro-porous materials with different properties is summarized in ref. 10. There exist also several reviews describing specific application driven requirements such as specific large absorption areas, high surface to volume ratios,^5,8,11^ and prospective developments.^12^ However, most methods of making porous polymer membranes are rather complex and often need very elaborate precision chemistry, and require processes controlled in detail.

In our recent work,^13–15^ we have demonstrated by simulation and by experiment that stable well controlled nanoporous films can be made just by mechanical deformation of highly entangled monodisperse polymer films and a subsequent quench of this nonequilibrium material into the glassy state. We have shown that the long chains extend over several bridges between pores. The relaxation is significantly slowed down by chain entanglements, which display a high density at regions of merging bridges between pores. This stabilizes the pores and prevents further growth and coalescence. In our previous study we have found typical pore diameters *d*_p_ ≈ 5–10*d*_*T*_, *d*_*T*_ being the reputation tube diameter. For the simulation part we considered a melt of chains of length *N* = 2000 ≈ 71*N*_e_, *N*_e_ = 28 being the entanglement length while for experiment we used polystyrene (PS) of 1000 kDa ≈ 60*M*_e_ with a polydispersity index of *M*_W_/*M*_N_ = 1.03. Thus in both cases there were either no short chains or their volume fraction was negligible. Of course if one thinks of a broader application of such a process, which is just based on mechanical deformation of a pure polymer system without any additives or stabilizing chemical reactions, the demand of such a high monodispersity could be detrimental. Thus we investigate the influence of short chains on the properties of the mechanically deformed polymer films by studying a system containing an equal number of long and short chains. We show that the same process can be successfully applied to such polydisperse highly entangled polymer films. The short chains aggregate in the center of the polymer bridges, which form the pore walls and avoid contact with the surface. By that the average pore size is somewhat larger due to a slightly increased effective entanglement length, but the qualitative picture remains unchanged.

The outline of the paper is as follows: we first describe the system and investigate the morphological properties of polydisperse film subject to biaxial expansion in Section II. Then the developments of porous structures of thin expanded films upon subsequent relaxation in Section III and cooling in Section IV are analyzed. Finally, Section V contains our conclusions.

## II. Morphology of polydisperse films upon biaxial deformation

We consider a thick polymer film containing an equal number of chains of two lengths, *N*_1_ = 1900 and *N*_2_ = 100, respectively, which leads to a polydispersity index of *M*_W_/*M*_N_ = 1.8. This system was prepared by starting from the fully equilibrated free-standing monodisperse polymer film containing 1000 weakly semiflexible chains of *N* = 2000 monomers of a simulation model based on a modification of the standard bead-spring model,^16–19^ which is well suited to study systems with free surfaces and the glass transition, at bulk melt (monomer number) density^14,20^*ρ*_0_ = 0.85*σ*^−3^. To prepare the bidisperse films a short piece of length *N*_2_ = 100 was cut off at the end of each chain of *N* = 2000. This film of now 2000 chains was equilibrated for about 3*τ*_R,*N*=*N*_2__, *τ*_R,*N*_ being the Rouse time of chains of size *N*, a time long enough for the short chains to move their own diameter. Alternatively, equilibrated polydisperse films can also be prepared *via* a soft-sphere approach and hierarchical backmapping.^20,21^ By this we arrived at eqilibrated films of effective thickness *h* ≈ 130*σ* ≈ 4.4*R*^(0)^_g_(*N* = *N*_1_) and two lateral dimensions with periodic boundary conditions, *L*_*w*_ = *L*_*x*_ = *L*_*y*_ ≈ 134*σ*. For comparison, *R*^(0)^_g_(*N* = *N*_1_ = 1900) ≈ 29.3*σ* and *R*^(0)^_g_(*N* = *N*_2_ = 100) ≈ 6.7*σ* are the root-mean square radii of gyration for bulk chains^22^ of size *N*, while we here have *R*_g_(*N*_1_ = 1900) ≈ 28.5*σ* and *R*_g_(*N*_2_ = 100) ≈ 6.7*σ*, showing that chain in thick films are very similar to those in bulk.^14,20^

This free standing film is subject to a simple “biaxial expansion” deformation. It is stretched into two lateral dimensions, *i.e.*, equal-biaxial strain^23–25^ with periodic boundary conditions up to a maximum expansion of *λ* × *λ* ≈ 4 × 4 at *T* = 1.0*ε*/*k*_B_, while the thickness of the film is free to adjust, *cf.* Fig. S1 of ESI† (ref. 26). We follow the same protocol as in our previous work.^13,14^ In our earlier work we compared fast and slow deformation and found no significant differences in the results. Thus, we here apply the fast deformation rate, as shown in Fig. S1 and S2 (ESI†). The deformation rate is set that we can expect subchains of length of up to 0.6*N*_e_ ≈ 17 can equilibrate during the expansion, while the conformations of longer strands will be affected. Details are given in the appendix.

**Fig. 1:**
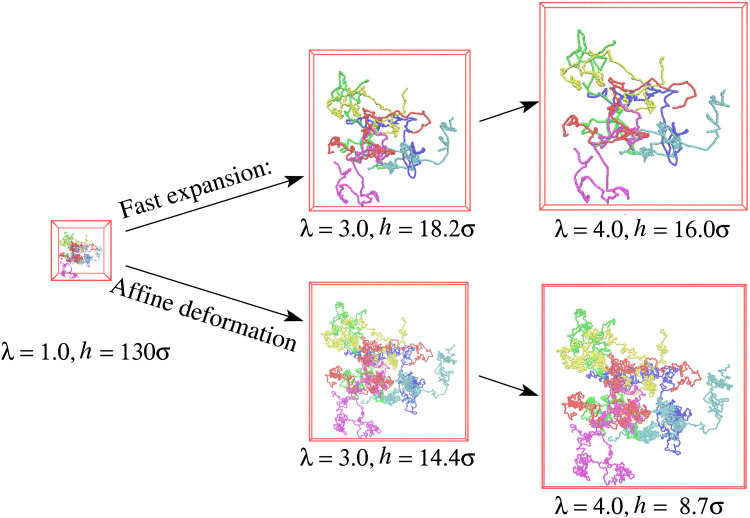
Snapshot configurations of six selected chains of *N*_1_ = 1900 out of the free-standing polydisperse film (upper) and hypothetical affine deformation (lower) of the initial conformation at *λ* = 1.0, 3.0, and 4.0, as indicated.

Snapshots of free-standing polydisperse polymer films for *λ* ≈ 1.0, 3.0, and 4.0 produced with an effective average strain rate *ε̇τ*_e_ ≈ 2.61 (see Fig. S2b, ESI†) are shown in Fig. 1. To demonstrate the conformational changes of long chains in the film, six randomly selected chains of *N*_1_ = 1900 are marked in different colors. Obviously, the overall shape of long chains follows an affine deformation while short chains do not (shown in Fig. S3 of ESI†). The overall morphologies are quite similar as they were observed for a monodisperse polymer films subject to a similar expansion rate *ε̇τ*_R,*N*=2000_ ≈ 14 710, *i.e.*, *ε̇τ*_e_ ≈ 2.88, also allowing for relaxation of subchains up to about 0.6*N*_e_ (see ref. 14).

**Fig. 2:**
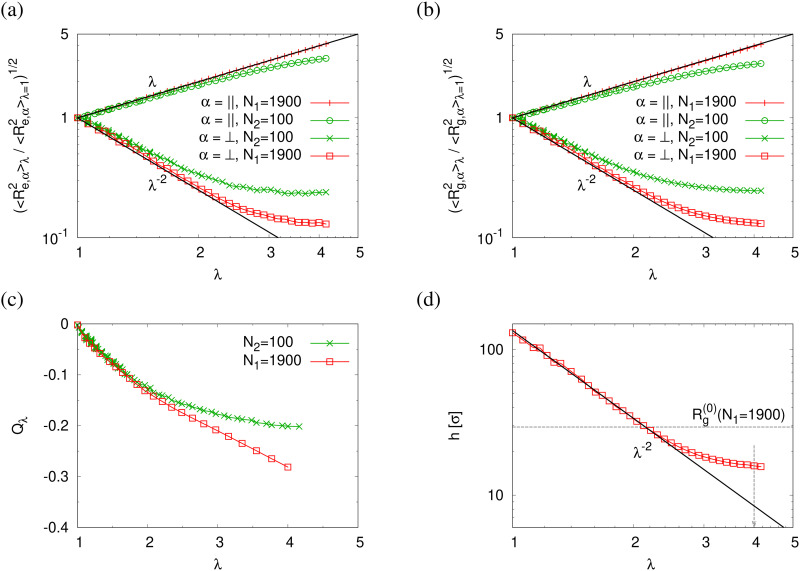
Two components of rescaled root-mean-square (rms) end-to-end distance, (〈*R*_e,*α*_^2^(*N*)〉_*λ*_/〈*R*_e,*α*_^2^(*N*)〉_*λ*=1_)^1/2^ (a) and rescaled rms radius of gyration, (〈*R*_g,*α*_^2^(*N*)〉_*λ*_/〈*R*_g,*α*_^2^(*N*)〉_*λ*=1_)^1/2^, bond orientation order parameter *Q*_*λ*_ (c), and effective film thickness *h* (d), plotted *versus* the strain of *λ*. In (a) and (b), *α* = ‖ and ⊥ denote the components in the direction parallel and perpendicular to the expanding directions, respectively. In (a)–(c), data for two different chain lengths *N*_1_ = 1900 and *N*_2_ = 100 in expanded polydisperse film are shown, as indicated. Reference values are 〈*R*_e,‖_^2^(*N*_1_ = 1900)〉_*λ*=1_ ≈ 3567*σ*^2^, 〈*R*_e,⊥_^2^(*N*_1_ = 1900)〉_*λ*=1_ ≈ 1082*σ*^2^, 〈*R*_e,‖_^2^(*N*_2_ = 100)〉_*λ*=1_ ≈ 180*σ*^2^, 〈*R*_e,⊥_^2^(*N*_2_ = 100)〉_*λ*=1_ ≈ 85*σ*^2^ in (a), and 〈*R*_g,‖_^2^(*N*_1_ = 1900)〉_*λ*=1_ ≈ 599*σ*^2^, 〈*R*_g,⊥_^2^(*N*_1_ = 1900)〉_*λ*=1_ ≈ 211*σ*^2^, 〈*R*_g,‖_^2^(*N*_2_ = 100)〉_*λ*=1_ ≈ 30*σ*^2^, 〈*R*_g,⊥_^2^(*N*_2_ = 100)〉_*λ*=1_ ≈ 14*σ*^2^ in (b). In (a), (b) and (d), affine scaling laws are shown by straight lines for comparison.

Normalized strain-dependent chain extensions are shown in Fig. 2a and b. Affinely, the deformation follows *λ* for the parallel components and *λ*^−2^ for the perpendicular, respectively. For *N*_1_ = 1900 the in-plane expansion is affine all the way up to *λ* ≈ 4.0, while deviations for *N*_2_ = 100 develop around *λ* ≈ 2.6. In the perpendicular direction, long chains deform affinely (*λ*^−2^) only up to *λ* ≈ 2.4 in agreement with the adjustment of the film thickness, *cf.*Fig. 2d, while short chains deform nonaffinely almost from the very beginning. These global conformational changes also lead to characteristic changes in the bond orientational order parameter *Q*_*λ*_. Choosing the *z*-axis as a reference, 
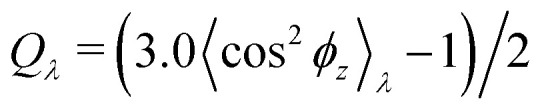
 where *ϕ*_*z*_ is the angle between any bond vector and the *z*-axis. For an isotropic distribution of bond directions *Q*_*λ*_ = 0, while *Q*_*λ*_ = −1/2, if all bonds would lie in the *xy* plane. The strain-dependent orientational order parameter *Q*_*λ*_ of bond vectors along chains of *N*_1_ = 1900 shows that bond vectors in the extended films tend to lie randomly along the direction parallel to the interfaces. For short chains of *N*_2_ = 100, *Q*_*λ*_ approaches a plateau value for *λ* > 3.5. The effective film thickness *h* determined from the monomer density profile (see Fig. S4a of ESI†) follows affine deformation up to *λ* ≈ 2.6 then approaches a plateau value approximately for *λ* ≥ 3 in the thin film regime, Fig. 2d.

**Fig. 3:**
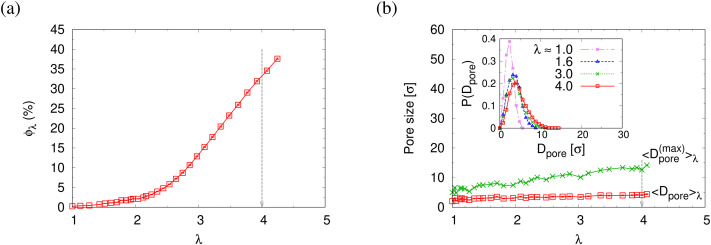
Porosity *ϕ*_*λ*_ (a), pose size characterized by the mean pore size 〈*D*_pore_〉_*λ*_ and mean maximum pore size 〈*D*^(max)^_pore_〉_*λ*_ (b), plotted as a function of strain *λ* for polydisperse film subject to expansion. Pore size distributions *P*(*D*_pore_) plotted as a function of *D*_pore_ at several selected strain values of *λ* are shown in the inset of (b).

The reduction of the normalized monomer density profile for *λ* > 2.3 shown in Fig. S4a (ESI†) indicates the onset of porosity *ϕ* (see Fig. 3a). Considering monomers in long and short chains separately, Fig. S4b and c show that *ρ*_1_(*z*) and *ρ*_2_(*z*) follow the same behavior as *ρ*(*z*). Here the porosity *ϕ* and pore size distribution *P*(*D*_pore_) of pore diameter *D*_pore_ in expanded films is estimated following the definition given by Gubbins *et al.*,^27–30^ where *ϕ* and *P*(*D*_pore_) depend on the accessible volume of a hard spherical test particle of size 1.0*σ*. *I.e.* the test particles explore all regions in the film, where the nearest monomer is at least a distance of 1*σ* away. This is a purely geometrical measure, as no interaction between test particles and monomers is considered. The porosity *ϕ* is then given by the percentage of void volume *V*_void_ compared to the total effective volume *V*_film_ = *hL*_*x*_*L*_*y*_ of the films.^14^ Results of the strain-dependent porosity *ϕ*_*λ*_, average pore size 〈*D*_pore_〉_*λ*_, maximum pore size 〈*D*^(max)^_pore_〉_*λ*_, and probability distribution of pore size *P*(*D*_pore_) are shown in Fig. 3. The porosity *ϕ* increases monotonically with the increase of strain *λ*. No significant change in 〈*D*_pore_〉 while 〈*D*^(max)^_pore_〉 increases with the increase of strain *λ* slightly. *P*(*D*_pore_) has a unimodal-like distribution and the distribution becomes broader with the increase of *λ*. Again this is very similar to the results of monodisperse films.

**Fig. 4:**
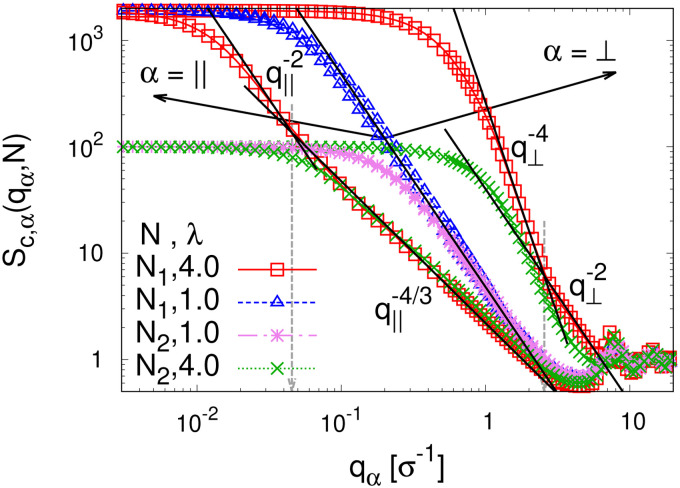
Two components of single chain structure factor *S*_c,*α*_(*q*_*α*_,*N*) in the directions parallel (*α* = ‖) and perpendicular (*α* = ⊥) to the expanding directions for chains of *N*_1_ = 1900 and *N*_2_ = 100 at *λ* = 1.0 and 4.0, as indicated. The theoretically predicted scaling laws are shown by straight lines for comparison, *cf.* text.

To detect the anisotropy of the chain structure and to compare short and long chains the strain-dependent two components of single chain structure factor *S*_c,‖_(*q*_‖_,*N*) and *S*_c,⊥_(*q*_⊥_,*N*) are shown in Fig. 4. Initially, at *λ* = 1 both, *S*_c,‖_(*q*_‖_,*N*) and *S*_c,⊥_(*q*_⊥_,*N*) ∼ *q*_⊥_^−2^ follow the Gunier law decay *S*_c,*α*_(*q*_*α*_,*N*) = *N*(1 − *q*_*α*_^2^*R*_g,*α*_^2^(*N*)/3) for small *q*_*α*_ as expected for ideal bulk chains. As the strain *λ* increases, *S*_c,⊥_(*q*_⊥_,*N*) increases while *S*_c,‖_(*q*_‖_,*N*) decreases which are consistent with the change in 〈*R*_g,⊥_^2^(*N*)〉_*λ*_ and 〈*R*_g,‖_^2^(*N*)〉_*λ*_ shown in Fig. 2b, respectively. In the thin film regime (*λ* ≥ 3.0), chains are highly stretched in the expansion plane. As for the monodisperse case chains *S*_c,‖_(*q*_‖_,*N*) ∼ *q*_‖_^−4/3^ in an intermediate *q*_‖_ range. We relate this 2-d self-avoiding walks like structure to the pore structure in the film, as argued below. Again there is no difference between short and long chains in this regime. As for the monodisperse case the pores seem to introduce an effective excluded volume on shorter and intermediate length scales. Only on large length scales, *q*_‖_ ≤ 0.045*σ*^−1^ the ideal chain behavior *S*_c,‖_(*q*_‖_,*N*) ∼ *q*_‖_^−2^ is recovered for *N* = *N*_1_ = 1900. In contrast, *S*_c,⊥_(*q*_⊥_,*N*) ∼ *q*_⊥_^−2^, on short length scales (*q*_⊥_ > 2*σ*^−1^). On large length scales, a sharp interface described by a Porod law like scaling *S*_c,⊥_(*q*_⊥_,*N*) ∼ *q*_⊥_^−4^ is observed for *λ* ≥ 3.0. However, the short chains seem to be a bit compressed in the perpendicular direction on shorter length scales.

We also calculate the two components of the collective scattering function of the films, *S*_‖_(*q*_‖_) and *S*_⊥_(*q*_⊥_), respectively (see Fig. S5 of ESI†). The intensity of *S*_‖_(*q*_‖_) increases with *λ* on large and intermediate length scales while on short length scales (*q*_‖_ > 2*σ*^−1^), it remains unchanged, showing that the local monomer packing is still conserved. The conserved peak at 
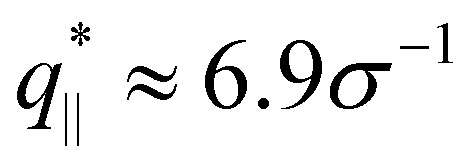
 shows that the inter-monomer packing distance of 
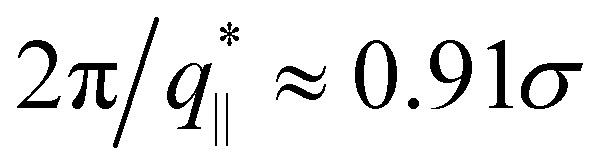
 still remains the same. On large and intermediate length scales, *S*_‖_(*q*_‖_) ∼ *q*_‖_^−2^ is observed at *λ* ≥ 3.0. The sharp local minima at *n*_*q*_ = *hq*_⊥_/(2π), *n*_*q*_ = 1, 2,… in the curves of *S*_⊥_(*q*_⊥_) confirm that the film thickness estimate *h* shown in Fig. 2d is consistent with the estimate from *S*_⊥_(*q*_⊥_). Even the *q*_⊥_^−4^ envelope for *λ* = 4 is reasonably well displayed, which is an indication of a rather uniform film thickness. Altogether, the data are almost indistinguishable from the monodisperse case. There is, however one interesting difference for *λ* = 1.0. The low *q*_‖_ regime indicates a slightly smaller compressibility or spatial density inhomogeneity for the polydisperse film compared to the monodisperse one. This indicates that short chain additions seem to level out fluctuations more effectively.

## III. Relaxation of expanded polydisperse film at *T* = 1.0*ε*/*k*_B_

**Fig. 5:**
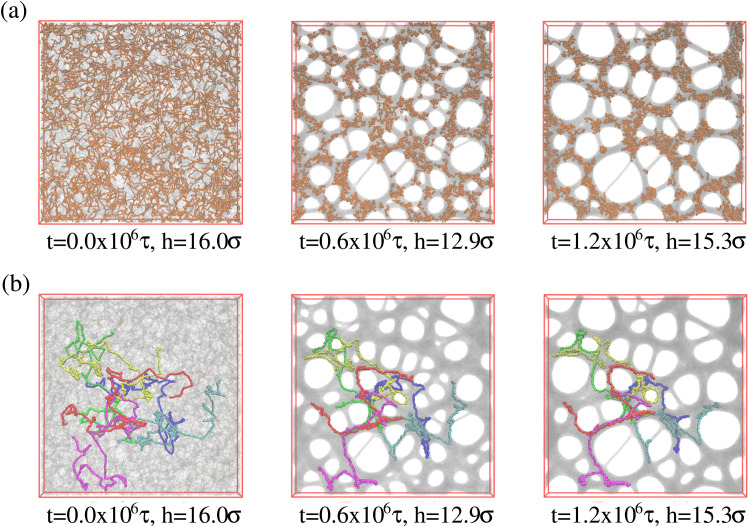
Snapshot configurations of thin polydisperse porous films at *λ* ≈ 4.0 subject to relaxation at several selected relaxation times *t* and assumed thicknesses *h*, as indicated where all 1000 chains of chain length *N*_2_ = 100 are marked in orange colors (a), and six randomly selected chains of chain length *N*_1_ = 1900 are marked in different colors (b).

So far we have studied the initial structure of the expanded films and found that the differences between mono- and polydisperse films are only marginal and that the dominance of the very long chains to determine the film properties remains unchanged. We now turn to the relaxation of the expanded film at the process temperature, kept at *λ* ≈ 4.0. Our previous study on monodisperse films at *λ* ≈ 4.0 has shown a strong retardation of conformational relaxation. This eventually stabilized nanoscopic pores, which then slowly grew under fixed strain. The overall pore diameter was limited by entanglement constraints. The central question here is, whether and how the shorter chains influence the stability and growth of pores. It should be kept in mind that *T* = 1.0*ε*/*k*_B_ ≈ 1.5*T*^(0)^_g_, *T*^(0)^_g_ ≈ 0.67*ε*/*k*_B_ being the glass transition temperature of a bulk polymer melt.^31,32^ To investigate the influence of polydispersity we follow the same relaxation protocol as in ref. 13 and 14 and observe the relaxation of the films by MD simulations in the NVT ensemble over a time of up to *t* = 1.2 × 10^6^*τ*. Fig. 5 shows the morphological changes of expanded polydisperse polymer film, see Fig. S6 in the ESI,† for more details. We observe the same nucleation and slowed coarsening of the hole structures as for the monodisperse case with, however, somewhat larger holes. There is one important difference between a typical nucleation and the growth mechanism in our present system. Small pores, which disappear by shrinking and not by merging with other pores, since the long polymers extend over several holes merging is prevented. Fig. 5 already points to three important features. The first, just as for the monodisperse films, long chains extend over several pores, stabilizing the whole morphology. Second, the short chains seem to tend to aggregate inside the polymer matrix and avoid the surface regions. This is even better seen, when we look at thin slices of the film. Slices of thickness 3*σ* are shown in Fig. S7 in the ESI.† And third, this aggregation scenario is very similar to strain-induced segregation between the strained long and relaxed short chains is observed due to the reduction of conformational constrains of short chains imposed by the surrounding.

Times covered range up to *t* = 1.2 × 10^6^ ≈ 530*τ*_e_, corresponding to the Rouse time of subchains of length of *N*_s_ ≈ 644. Similarly, as observed for monodisperse films upon fast expansion and in contrast to slow expansion, initially no well defined pore structure is seen. After a short time well defined pores nucleate. The pore sizes increase accompanied by some drop in the number of pores. The growth of the pores slows down significantly after about half of the relaxation time, which corresponds to the Rouse time of subchains of about 16*N*_e_. Pore sizes in the fast expanded monodisperse film appear only weakly smaller than in the polydisperse film. This relaxation retardation is also demonstrated by the six marked chains whose conformations only marginally change, obviously due to the topological constraints these highly entangled chains encounter.^33–35^ Moreover, in all cases the shape of pores tends to become spherical to minimize surface tension.

**Fig. 6:**
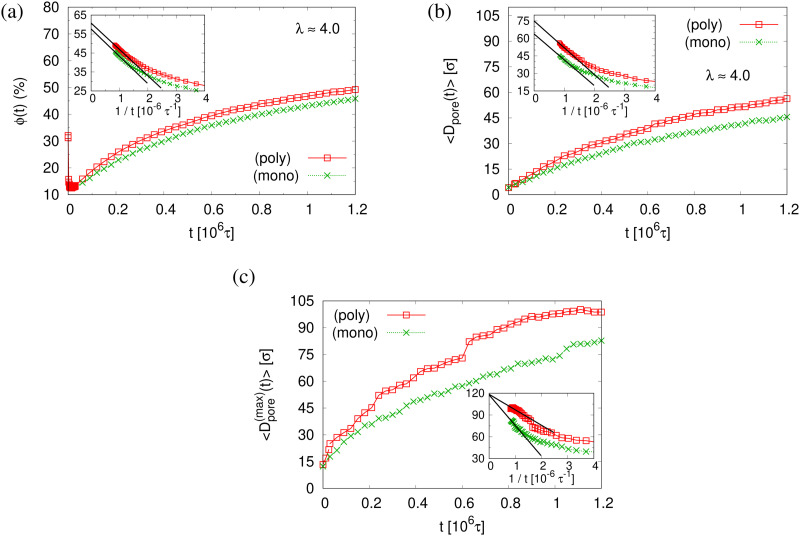
Porosity *ϕ*(*t*) (a), mean pore size 〈*D*_pore_(*t*)〉 (b), and mean maximum pore size 〈*D*^(max)^_pore_(*t*)〉 (c), plotted *versus* relaxation time *t* for polydisperse (poly) and monodisperse (mono) porous films at *λ* ≈ 4.0, as indicated. In the inset of (a)–(c), we plot the same data for *t* > 0.25 × 10^6^*τ*, respectively, *versus* 1/*t*. The straight lines indicate linear extrapolating of all data sets to *t* → ∞.

**Fig. 7:**
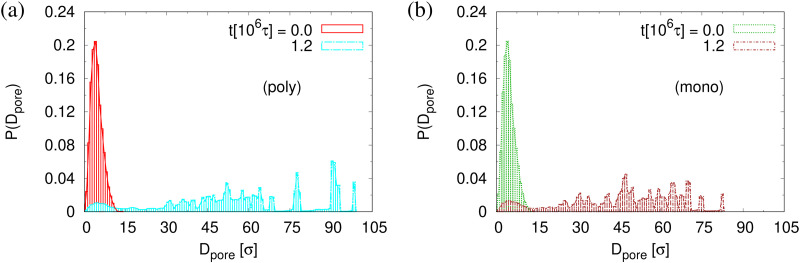
Histogram plot of pore size distribution *P*(*D*_pore_) for thin polydisperse (a) and monodisperse (b) porous films at *λ* ≈ 4.0. Only data for thin porous films at the subsequent relaxation times *t*/*τ* = 0 and 1.2 × 10^6^ are shown, as indicated.

The small difference in pore size and porosity is confirmed by the direct measurement of the porosity *ϕ*(*t*), the pore size *D*_pore_(*t*), and the maximum pore size *D*^(max)^_pore_(*t*) which all increase upon time (see Fig. 6). For both films the average pore sizes and maximum pore sizes continue to grow only very slowly with increasing time. The average pore diameter at *t* = 1.2 × 10^6^*τ* corresponds to about 46*σ* ≈ 9*d*_*T*_ for monodisperse film. Here we observe 56*σ* for the polydisperse film. Assuming that this size is directly determined by the tube diameter, we estimate the shift in *d*_*T*_ upon cutting off the short chains, since they have ample time to relax completely in the course of the relaxation. With *N*_1_ = 1900 for the polydisperse system the packing length *p* = *N*_1_/(*ρ*_1_*R*^2^(*N*_1_)) is increased by a factor of 1/0.95, *ρ*_1_ being the density of just the long chains. Taking the relation between plateau modulus and *p* and *N*_e_, respectively, one would expect a shift of *d*_*T*_ of about 5%, which is relatively close to the shift in the pore diameter.^36^ Assuming a linear extrapolation to 1/*t*→0, *ϕ*(*t*) ≈ 61% and 〈*D*^(max)^_pore_(*t*)〉 ≈ 120*σ* converge within error to the same values for both cases while 〈*D*_pore_(*t*)〉 is larger for expanded polydiseperse film, 〈*D*_pore_(*t*)〉 ≈ 75*σ* ≈ 14*d*_*T*_, and 64*σ* ≈ 13*d*_*T*_ for the monodisperse film, respectively. Again this shift is roughly within the expected range. A more detailed theoretical account, however, is needed to make this argument more quantitative. The probability distributions of pore size *D*_pore_, *P*(*D*_pore_) are shown in Fig. 7. At *t* = 0*τ*, *P*(*D*_pore_) has a unimodal distribution for both cases, and it becomes a much broader multimodal distribution at *t* = 1.2 × 10^6^*τ*. For polydisperse film, results of *P*(*D*_pore_) show that the probability of finding larger pore size *D*_pore_ increases while it decreases for small pore sizes, as illustrated in Fig. 5, similar as for the expanded monodisperse film upon fast expansion.

**Fig. 8:**
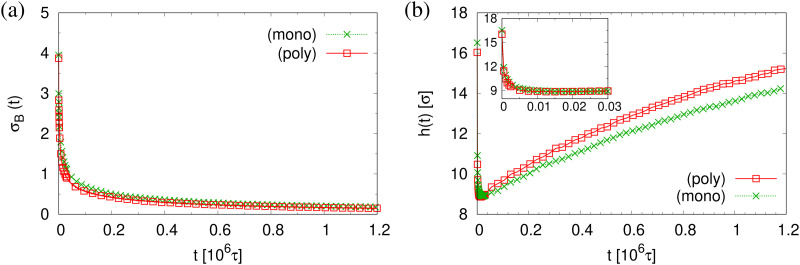
Net stress *σ*_B_(*t*) (a), and film thickness *h*(*t*) (b), plotted *versus* relaxation time *t* for polydisperse and monodisperse porous films at *λ* ≈ 4.0.

**Fig. 9:**
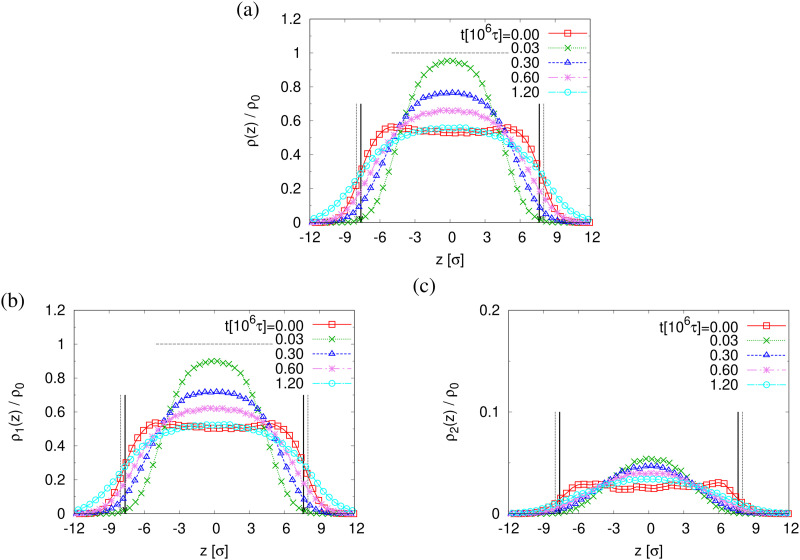
Rescaled monomer density profiles for all monomers, *ρ*(*z*)/*ρ*_0_ (a), monomers in chains of *N*_1_ = 1900, *ρ*_1_(*z*)/*ρ*_0_ (b), and *N*_2_ = 100, *ρ*_2_(*z*)/*ρ*_0_ (c), plotted as a function of *z* at several selected subsequent relaxation times *t*, as indicated. The centers of films in the *z*-direction are matched at *z* = 0*σ*. The interfaces located at *Z*^(lower)^_*G*_ and *Z*^(upper)^_*G*_ determined from *ρ*(*z*) for films at *t* = 0*τ* and 1.2 × 10^6^*τ* are indicated by dashed and solid arrows, respectively.

The finding that the system relaxation slows down significantly also is supported by the reduction of restoring forces per unit area, *σ*_B_(*t*), shown in Fig. 8. We observe a dramatic reduction in net restoring stress *σ*_B_(*t*) = |*P*_*zz*_(*t*) − (*P*_*xx*_(*t*) + *P*_*yy*_(*t*))/2| after a very short initial time of about (0.2–0.3) × 10^6^*τ*. At the same time *P*_*zz*_(*t*) remains at 0.0*ε*/*σ*^3^. Furthermore, after that the time-dependent stress is almost indistinguishable between these two cases, again in accord with the visual inspections of the membranes. The results of *h*(*t*) in Fig. 8b show that the initial reduction of film thickness for both cases follows the same curve. This reverse behavior for the films upon fast expansion also appears in the change of monomer density profile *ρ*(*z*) (see Fig. 9). Eventually the expanded polydisperse film upon relaxation remains somewhat thicker in agreement with the apparently slightly larger porosity and thus larger pores.

**Fig. 10:**
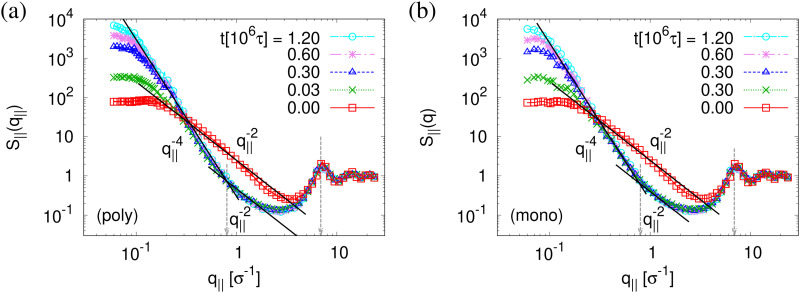
Collective structure factor *S*_‖_(*q*_‖_) in the expanding direction for expanded thin polydisperse (a) and monodisperse (b) films at *λ* ≈ 4.0, plotted *versus* the wave factor *q*_‖_ at several selected relaxation times *t*, as indicated. Theoretical predictions are shown by solid straight lines for comparison.

The above described scenario is well supported by the in expansion plane collective structure factor *S*_‖_(*q*_‖_) in Fig. 10 at several relaxation times *t*. The region around the amorphous halo at 
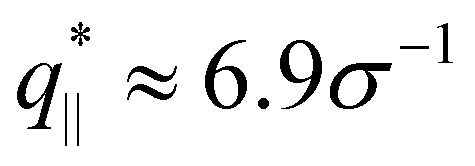
 remains unchanged upon relaxation for all times. Thus the local bead packing is not affected by our processes. With the increase of time, the signature of sharp pore surfaces, Porod scaling *S*_‖_(*q*_‖_) ∼ *q*_‖_^−4^, is stabilized and extended a little further to larger length scales, as expected by the slow increase of porosity^37^*ϕ*(*t*) (see Fig. 6). For both cases, the initially fuzzy interfaces sharpen and already after short relaxation time the same scaling is observed. The initially large *q*_‖_^−2^ regime narrows down to a small region. In all cases, *S*_‖_(*q*_‖_) reaches a shallow maximum/plateau at low *q*_‖_, roughly corresponding to distances around 100*σ*, corresponding to 2–3 average pore diameters and reminding of a semidilute 2-d liquid of hard disks (*i.e.* the pores). It should be noted that the late time data are almost indistinguishable from the same data obtained from films, which were expanded in a much slower process, see ref. 14.

**Fig. 11:**
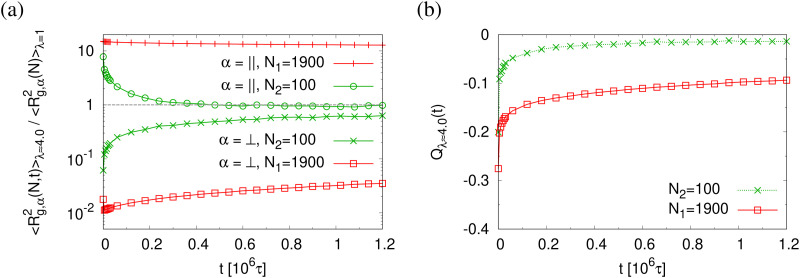
Two components of rescaled mean square radius of gyration 〈*R*_g,*α*_^2^(*N*,*t*)〉/〈*R*_g,*α*_^2^(*N*,*t* = 0)〉 (a) in the directions parallel (*α* = ‖) and perpendicular (*α* = ⊥) to the expending directions, and bond orientational order parameter *Q*_*λ*_(*t*) (b), plotted *versus* relaxation time *t*. Data are for two chain lengths *N*_1_ = 1900 and *N*_2_ = 100 in thin porous films at *λ* ≈ 4.0, as indicated.

**Fig. 12:**
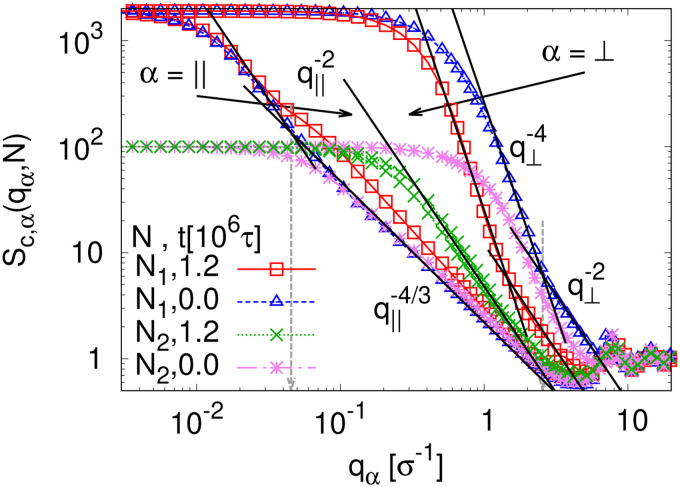
Two components of single chain structure factor *S*_c,*α*_(*q*_*α*_,*N*) in the directions parallel (*α* = ‖) and perpendicular (*α* = ⊥) to the expanding directions for two chain lengths *N*_1_ = 1900 and *N*_2_ = 100 in the expanded films. Data for several selected relaxation times *t* are shown, as indicated. Expected scaling laws are shown by straight lines for comparison, *cf.* text.

This comes along with the restricted conformational relaxation of the individual chains, as characterized by their linear dimensions and structure factor. The time-dependent two components of 〈*R*_g,*α*_^2^(*N*,*t*)〉 parallel (*α* = ‖) and perpendicular (*α* = ⊥), and the bond orientational order parameter *Q*_*λ*_(*t*) are shown in Fig. 11. For long chains (*N* = *N*_1_) 〈*R*_g,‖_^2^(*N*,*t*)〉 only decreases marginally during initial relaxation and then remains almost unchanged throughout the whole relaxation time. 〈*R*_g,⊥_^2^(*N*,*t*)〉 increases slightly with time *t* after a very short time initial decrease, but then gets stuck at a value compatible with the thickness of the thin film. Note that marginal relaxation indicates that the chain retraction inside the tube as predicted by the Doi-Edwards and GLaMM tube models^38,39^ here is strongly retarded. For short chains (*N* = *N*_2_), chain retraction is not observed since chains are only weakly entangled.^34^ 〈*R*_g,‖_^2^(*N*,*t*)〉 decreases while 〈*R*_g,⊥_^2^(*N*,*t*)〉 increases during initial relaxation and eventually move towards the equilibrium value. Especially 〈*R*_g,‖_^2^(*N*,*t*)〉 reaches the unperturbed bulk value. Similarly the bond orientational order parameter *Q*(*t*) displays a significant relaxation delay towards the isotropic phase for long chains while an isotropic distribution of bond directions is found for short chains. The two components of the single chain structure factor, *S*_c,‖_(*q*_‖_,*N*) and *S*_c,⊥_(*q*_⊥_,*N*), only change slightly with time *t* for *N* = *N*_1_ while for *N* = *N*_2_, *S*_c,‖_(*q*_‖_,*N*) ≈ *S*_c,⊥_(*q*_⊥_,*N*) ∼ *q*_⊥,‖_^−2^ at *t* = 1.2 × 10^6^ ≈ 42*τ*_R,*N*_1__, *cf.*Fig. 12, as expected for ideal chains. The instantaneously observed crossover for *N* = *N*_1_ from a two dimensional self-avoiding walk like structure (*S*_c,‖_(*q*_‖_,*N*) ∼ *q*_‖_^−4/3^) to ideal random walk like structure (*S*_c,‖_(*q*_‖_,*N*) ∼ *q*_‖_^−2^) at low *q*_‖_ is even shifted to lower *q*_‖_ and hardly visible anymore. However, the perpendicular component still displays a pronounced Porod power law (*S*_c,⊥_(*q*_⊥_,*N*) ∼ *q*_⊥_^−4^) indicating the sharp surface.

## IV. Stabilization by cooling

**Fig. 13:**
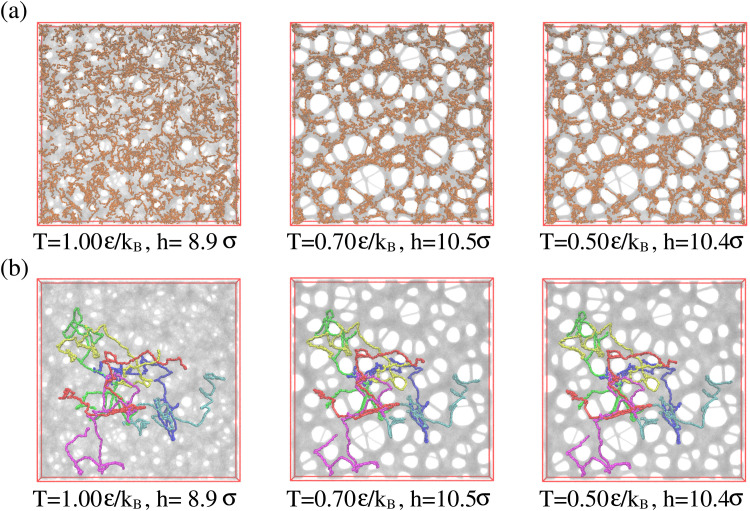
Snapshot configurations of free-standing thin polydisperse porous films at several selected temperatures *T*, as indicated where all 1000 chains of chain length *N*_2_ = 100 are marked in orange colors (a), and six randomly selected chains of chain length *N*_1_ = 1900 are marked in different colors (b).

Above we have shown, that the conformational and morphological relaxation of the expanded films is significantly retarded. The same and ultimate stabilization can be achieved by cooling the systems down towards the glass transition temperature *T*_g_.^32,40^ For this we follow a stepwise cooling protocol with the fixed cooling rate *Γ* = 8.3 × 10^−7^*ε*/(*k*_B_*τ*) = Δ*T*/Δ*t* (Δ*T* = 0.025*ε*/*k*_B_, Δ*t* = 30 000*τ*) by NVT MD simulations with Langevin thermostat, just as also applied for the monodisperse case.^13,14^ We apply this to the polydisperse film of film thickness *h* ≈ 16.0*σ* at *λ* ≈ 4.0, right after deformation. For this cooling rate chains of length *O*(100) can full relax around and weakly below *T* = 1.0*ε*/*k*_B_. Thus the short chains of *N*_2_ = 100 can equilibrate completely while for *N*_1_ = 1900 only subchains of similar lengths can relax. Fig. 13 illustrates this. Similar structures are observed for the thin monodisperse polymer film upon fast expansion.^14^ However, the pore sizes are slightly larger in the polydisperse system. The clustering of the short chain indicates some strain-induced segregation between the strained long chains and relaxed short chains. At the same time short chains seem to avoid the surfaces. However, this does not affect the suitability of the resulting porous polydisperse film, since long chains extend over several pore envelopes.

**Fig. 14:**
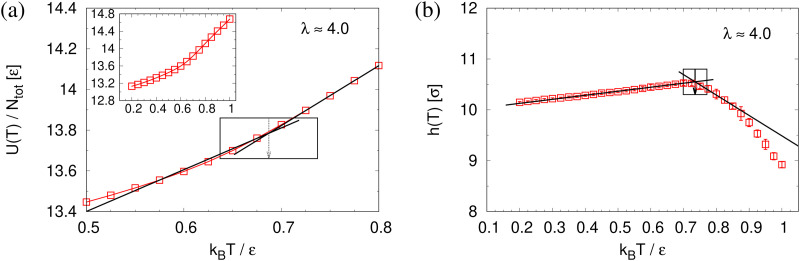
Total potential energy per monomer *U*(*T*)/*N*_tot_ (a) and film thickness *h*(*T*) (b) plotted as a function of temperatures *T* for expanded polydisperse film at *λ* ≈ 4.0. The linear lines give the best fit of our data along the liquid and the glass branch. The corresponding *T*_g_ is marked by an arrow. The whole set of data of *U*(*T*) is shown in the inset of (a).

**Fig. 15:**
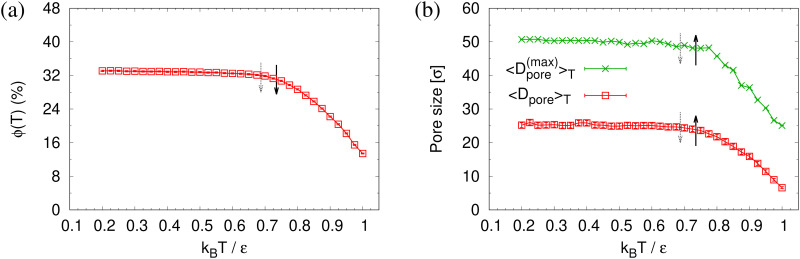
Porosity *ϕ*(*T*) (a), mean pore size 〈*D*_pore_〉_*T*_ and mean maximum pore size 〈*D*^(max)^_pore_〉_*T*_ (b), plotted as a function of temperature *T* for expanded thin polydisperse films at *λ* ≈ 4.0. Estimates of *T*_g_ from *h*(*T*) and *U*(*T*) are indicated by solid black and dotted gray arrows, respectively, *cf.*Fig. 14.

**Fig. 16:**
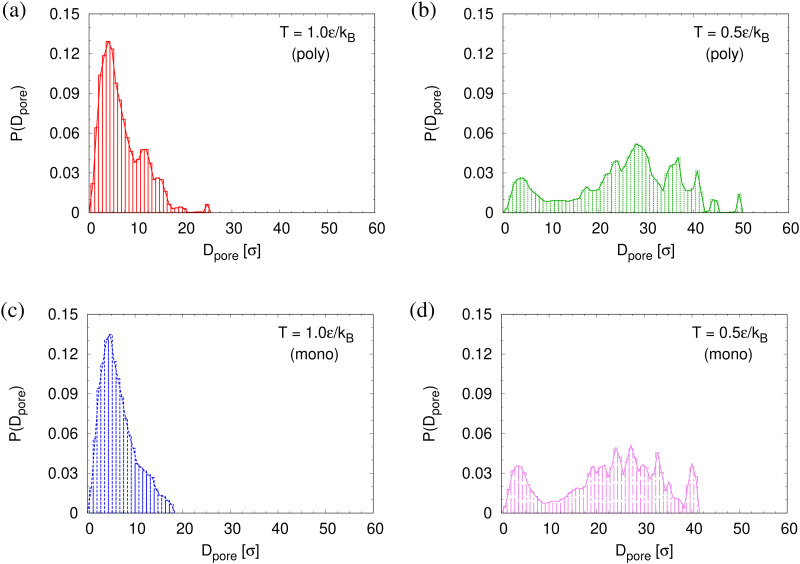
Histogram plot of pore size distribution *P*(*D*_pore_) at *T* = 1.0*ε*/*k*_B_ (a) and (c) and 0.5*ε*/*k*_B_ (b) and (d) right after relaxing for 30 000*τ* at each *T* for thin porous polydisperse (a) and (b) and monodisperse (c) and (d) films at *λ* ≈ 4.0 upon fast expansion subject to cooling.

To determine the apparent glass transition temperature *T*_g_ we resort to the total potential energy *U*(*T*) shown in Fig. 14, which gives *T*^*U*^_g_ = 0.69(5)*ε*/*k*_B_. A good measure of the density shift upon cooling is difficult due to the pore structure. Another estimate of *T*_g_ from the change in film thickness *h*(*T*) based from the monomer density profile (see Fig. S8, ESI†) gives *T*^*h*^_g_ = 0.74(4)*ε*/*k*_B_, respectively. The latter value is a bit too high, compared to independent studies of thin films.^32^ However, there is no influence of polydispersity on *T*_g_. The glass transition is around 0.70*ε*/*k*_B_ as found for thin monodisperse nanoporous films at ref. 14*λ* ≈ 4.0. The porous structures characterized by porosity *ϕ*(*T*) and by pore size *D*_pore_(*T*) are presented in Fig. 15. As *T* decreases *ϕ*(*T*) is increasing, approaching a plateau value in a temperature region, where also the monomer density profile *ρ*(*z*) converges. Both 〈*D*_pore_(*T*)〉 and 〈*D*^(max)^_pore_(*T*)〉 behave similar as *ϕ*. They all first increase with the decrease of *T* for *T* > *T*_g_, and then tend to reach a plateau approximately around *T*_g_. The resulting porosity *ϕ* ≈ 33%, 〈*D*_pore_〉 ≈ 25*σ*, and 〈*D*^(max)^_pore_〉 ≈ 50*σ* for *T* < *T*_g_ are slightly larger than the expanded monodisperse polymer film upon fast expansion (*ϕ* ≈ 29%, 〈*D*_pore_〉 ≈ 22*σ*, 〈*D*_max_〉 ≈ 42*σ*). The pore size distribution *P*(*D*_pore_) presented in Fig. 16 at *T* = 1.0*ε*/*k*_B_ is no longer a unimodal-like distribution after short chains are relaxed. At *T* = 0.5*ε*/*k*_B_, the multmodal distribution is slightly broader comparing to the expanded monodisperse film upon fast expansion.

**Fig. 17:**
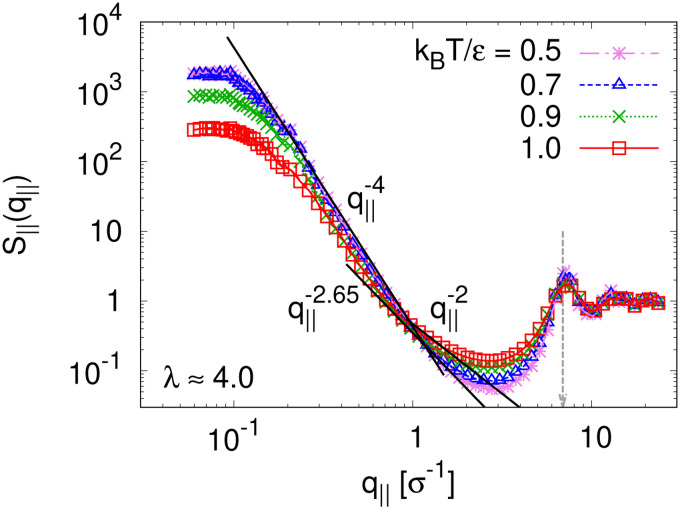
Collective structure factor *S*_‖_(*q*_‖_) in the expanding direction for expanded thin polydisperse film at *λ* ≈ 4.0, plotted *versus* the wave factor *q*_‖_ at several selected temperatures *T*, as indicated. Theoretical predictions are shown by solid straight lines for comparison.

The observed very strong similarity to the properties of monodisperse films also hold for the collective in-plane scattering function, *S*_‖_(*q*_‖_) as shown in Fig. 17. With the decrease of *T*, *S*_‖_(*q*_‖_) increases on large length scale, and levels off in a broad maximum/shoulder below *q* ≈ 0.2*σ*^−1^ related to the average pore size. More locally, surfaces become flat and sharp for *T* < *T*_g_, *i.e.*, *S*_‖_(*q*_‖_) ∼ *q*_‖_^−4^. The changes in local wall structure of pores and microscopic monomer packing also remains unchanged compared to the monodisperse example.

## V. Conclusions

In summary, we have extended the concept of employing entanglements to create and stabilize nanoporous polymer films^13,14^ to polydisperse melts. By choosing a bidisperse system of an equal number of very long (*N*_1_ = 1900) and short (*N*_2_ = 100) chains, *i.e. M*_W_/*M*_N_ = 1.8, the system contains short chains which can fully equilibrate throughout the film expansion process while the long chains cannot relax, leading to essentially conserved entanglements and by this to a stabilization of the pores. As in our first study, no additional chemical processing or stabilization beyond quenching the system into the glassy state is needed. Despite the addition of short chains even well above *T*_g_ the relaxation of the expanded film is significantly slowing down, making the whole process rather robust. The glass transition temperature itself for films at strain of *λ* ≈ 4.0 remains within our error bars at the bulk value. The temperature dependence of the film thickness, which displays a kink at a slightly higher temperature, points to additional stabilization mechanisms, which await a more detailed investigation. As in our previous study the pore properties are determined by the entanglement structure of the underlying polymer melt. However, the polydispersity leads to slightly larger pores of an extrapolated 〈*D*_pore_〉 ≈ 14*d*_*T*_, which is within the expected regime, considering faster relaxation and reduced entanglement density due to the shorter chains. Importantly, the short chains do not diffuse to the surfaces of the pores and thus do not fill up smaller pores. They stay within the bulk of the polymer matrix and by that can assume equilibrium conformations. The pores themselves act like an excluded volume interaction on the conformation of the chains winding around them, which is seen in the intermediate exponent in the scattering function. Altogether this study demonstrates that stable nanoporous polymer films can be made by mechanical deformation also for polydisperse systems as long as there are enough long chains which extend over several pores and by that stabilize the overall structure.

## Author contributions

H.-P. H. designed research, performed the molecular dynamics simulations, analyzed the data, and wrote the paper. K. K. designed research, analyzed the data and wrote the paper.

## Data availability

Data for this article are available on https://github.com/dehphsu/Publication_data/tree/b0d0357891fb1a7b0696163018f787479d28dac6/SM_2024.

## Conflicts of interest

There are no conflicts to declare.

## Supplementary Material

SM-020-D4SM00569D-s001

## Appendices

### Appendix

The components of pressure tensor along the two lateral dimensions are the same during the expansion process as shown in Fig. S2 (ESI†). Technically, the film is first instantaneously stretched by a factor of 1.02 along the *x*-direction, and then along the *y*-direction. This deformation step is chosen to be small enough, not to induce any numerical instabilities. After each deformation step along one direction the lateral dimensions are fixed and the system is allowed to relax for (0.02*τ*_e_/*C*), resulting in an effective strain rate *ε̇* = *C*/*τ*_e_. *C* is the measure of the strain rate relative to the entanglement time *τ*_e_ = *τ*_0_*N*_e_^2^ with the characteristic relaxation time^22^*τ*_0_ ≈ 2.89*τ* and the entanglement length^22,41^*N*_e_ ≈ 28. We initially use *C* = 6.27, *i.e.* the strain rate is much faster compared to the Rouse relaxation of the overall chains while subchains of chain lengths up to about  are expected to be able to relax during deformation. Moreover, after each expansion step the film thickness relaxes and the instant pressure *P*_*zz*_, quickly approaches zero as shown in Fig. S2 (ESI†). This latter relaxation required even a slowing down of the expansion leading to an effective *C* ≈ 2.61 based on the total time (2393.13*τ*) for deformation and thus leading to 0.6*N*_e_ ≈ 17, see ESI,† of ref. 14 for the detailed simulation protocol, and Fig. S2 (ESI†). This protocol is repeated until the desired expansion is reached. In our simulation, the expanded polydisperse polymer film finally is in the thin film regime (*h* ≈ 16.0*σ* < *R*^(0)^_g_(*N*_1_ = 1900)) with *P*_*zz*_ ≈ 0.0*ε*/*σ*^3^.
