# Supplementary material for: Stable polydisperse free-standing porous films made by mechanical deformation
Source: Soft Matter. 2024 Aug 2;20(34):6779–90. doi: 10.1039/d4sm00569d (PMC11322701; doi:10.1039/d4sm00569d)
Supplement: SM-020-D4SM00569D-s001 [file SM-020-D4SM00569D-s001.pdf]

Supplementary Information (SI) for Soft Matter.
This journal is © The Royal Society of Chemistry 2024

# Electronic Supplementary Information

# Stable polydisperse free-standing porous films made by mechanical deformation

Hsiao-Ping Hsu* and Kurt Kremer†

*Max-Planck-Institut für Polymerforschung, Ackermannweg 10, 55128, Mainz, Germany*

The time-dependent lateral dimensions $L_x(t)$, $L_y(t)$, film thickness $h$, and three diagonal components of pressure tensor, $P_{\alpha\beta}(t)$ with $\alpha = x$, $y$, and $z$ for the free-standing polydisperse film subject to biaxial expansion are shown in Figs. S1 and S2. The effective strain rate is estimated according to the definition $L_{x,y}(t)/L_{x,y}(0) = \exp(\dot{\varepsilon}t)$, see Fig. S2b.

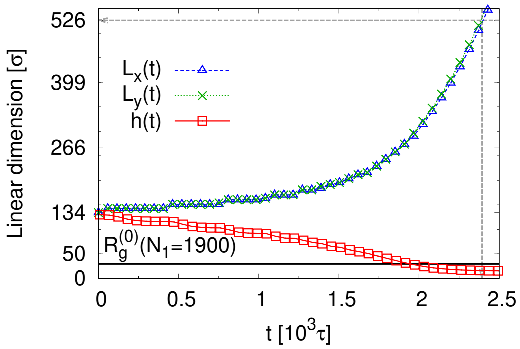


Figure S1. Time series of two lateral dimensions $L_x(t)$, $L_y(t)$, and thickness $h(t)$ of film for the film at $T = 1.0\epsilon/k_B$ upon biaxial expansion with $\dot{\varepsilon}\tau_e \approx 2.61$ (see Fig. S2b). Values of dimensions for the expansion ratio of $\lambda = L_{x,y}/L_w \approx 4.0$, i.e., $L_x(t) = L_y(t) \approx 526\sigma$ are indicated by arrows. Here $L_{x,y}(0) = L_w \approx 134\sigma$.

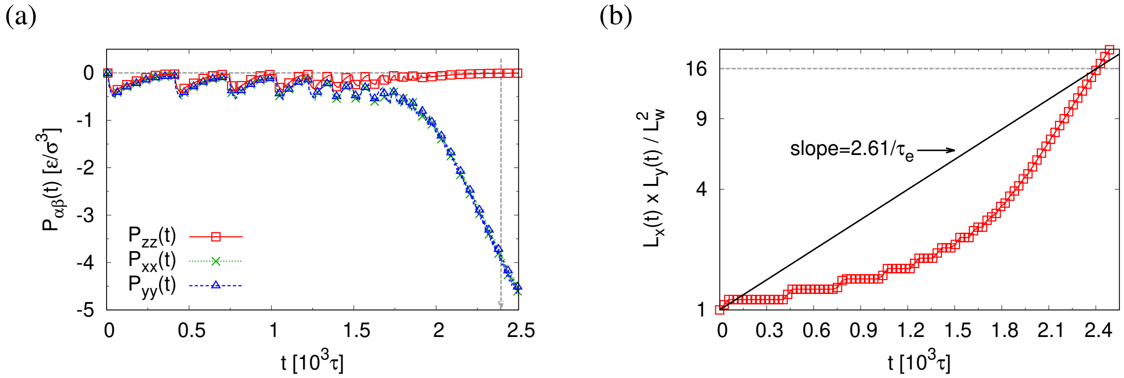


Figure S2. (a) Time series of three diagonal terms of pressure tensor $P_{\alpha\beta}$ for the film at $T = 1.0\epsilon/k_B$ upon biaxial expansion. (b) Change in lateral dimensions $(L_x(t) \times L_y(t))/L_w^2$ plotted versus the relaxation time $t$ on a semi-log scale. The effective strain rate $\dot{\varepsilon}$ is determined by the slope in (b). The expansion ratio of $\lambda = L_{x,y}/L_w \approx 4.0$, i.e., $L_x(t) = L_y(t) \approx 526\sigma$ is indicated by an arrow.

* hsu@mpip-mainz.mpg.de
† kremer@mpip-mainz.mpg.de

The conformational changes of six selected short chains of $N_2 = 100$ out of the free-standing polydisperse film do not follow an affine deformation as shown in Fig. S3.

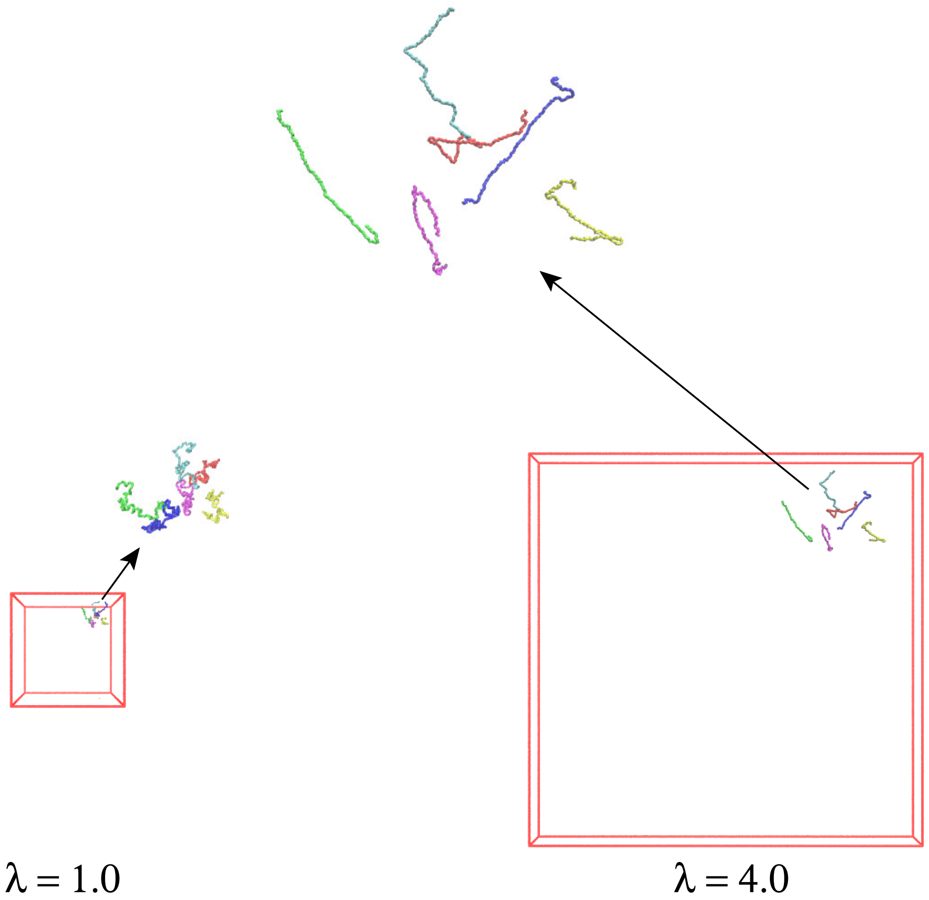


Figure S3. Six selected chains of $N_2 = 100$ out of the free-standing polydisper film at $\lambda = 1.0$ and 4.0 are shown as indicated.

The effective film thickness $h$ can either be estimated from the monomer density profiles $\rho(z)$, Fig. S4, or the scattering function in the direction perpendicular to the interfaces of films, Fig. S5.

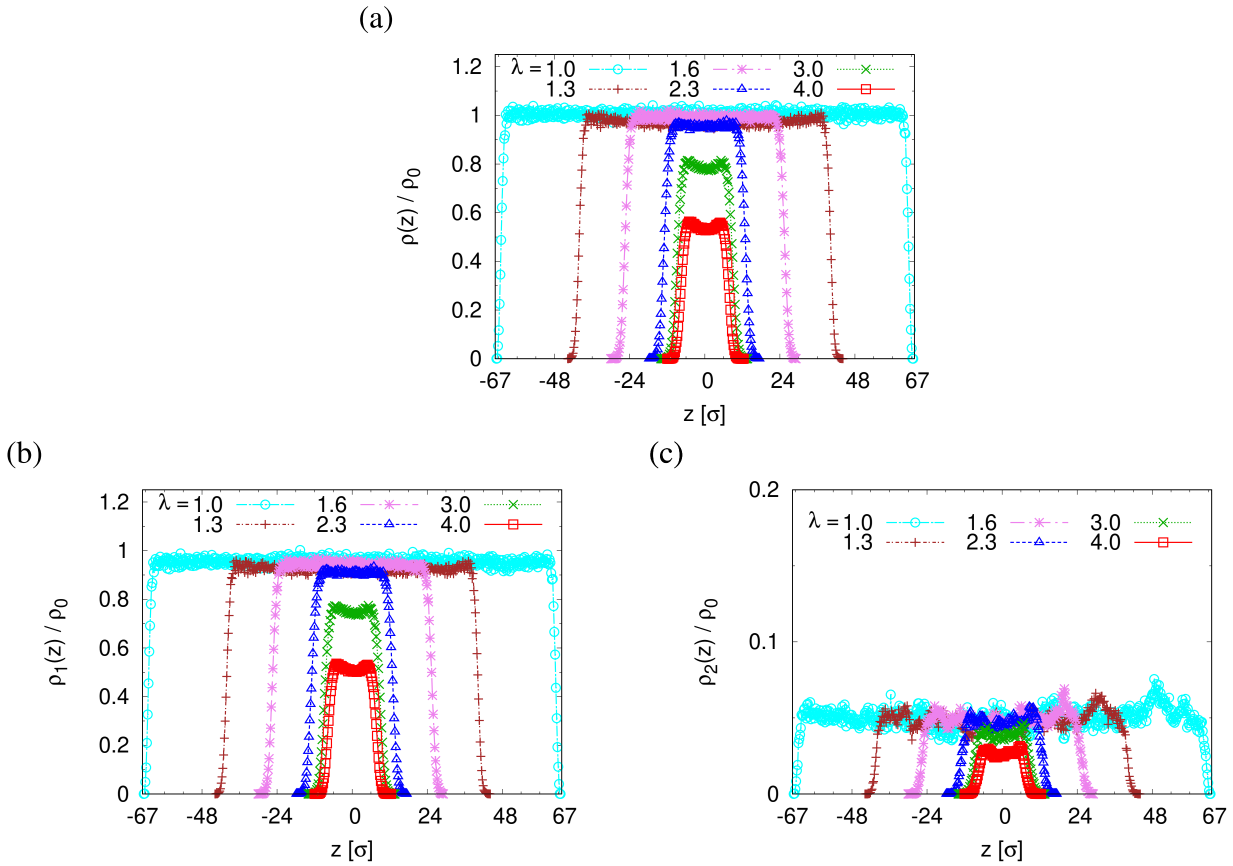


Figure S4. Monomer density profiles rescaled to the bulk melt density for all monomers, $\rho(z)/\rho_0$ (a), monomers in chains of $N_1 = 1900$, $\rho_1(z)/\rho_0$ (b), and $N_2 = 100$, $\rho_2(z)/\rho_0$ (c), respectively.

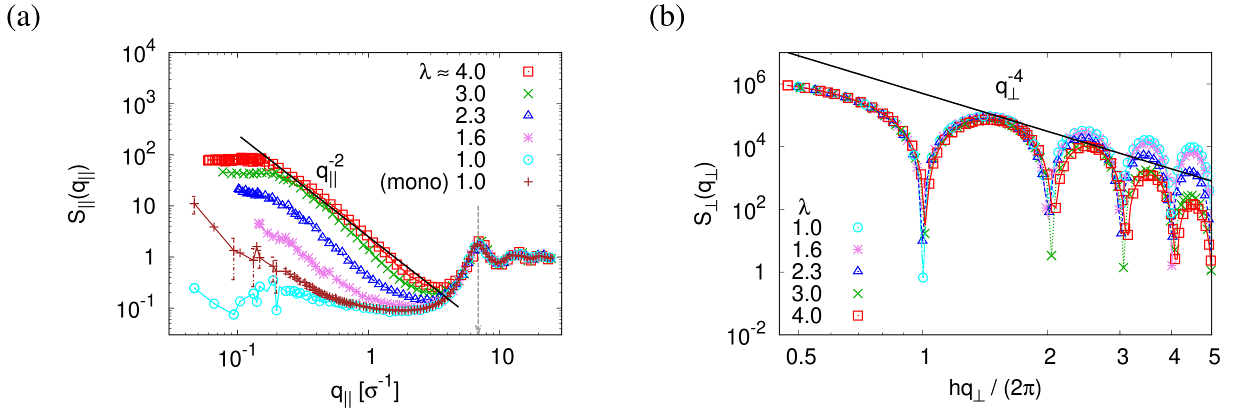


Figure S5. Two components of collective structure factor, $S_{||}(q_{||})$ (a) and $S_\perp(q_\perp)$ (b), in the directions parallel and perpendicular to the expanding direction, plotted versus $q_{||}$ (a), and $q_\perp/(2\pi/h(\lambda))$ (b). $S_{||}(q_{||}) \sim q_{||}^{-2}$ in (a), and the Porod law $S_\perp(q_\perp) \sim q_\perp^{-4}$ in (b) are also shown by straight lines for comparison. Data for an unperturbed monodisperse film at $\lambda = 1.0$ are included in (a), for comparison.

Time-dependent morphological changes of expanded thin polydisperse films at $\lambda \approx 4.0$ and $T = 1.0\epsilon/k_B$ indicating relaxation retardation are shown in Fig. S6. Detailed internal structures of films at $t = 0\tau$ and $1.2 \times 10^6\tau$ are investigated in Fig. S7. Fig. S8 shows the monomer density profile $\rho(z)$ at several selected temperatures $T$ for the expanded film at $\lambda \approx 4.0$ subject to cooling.

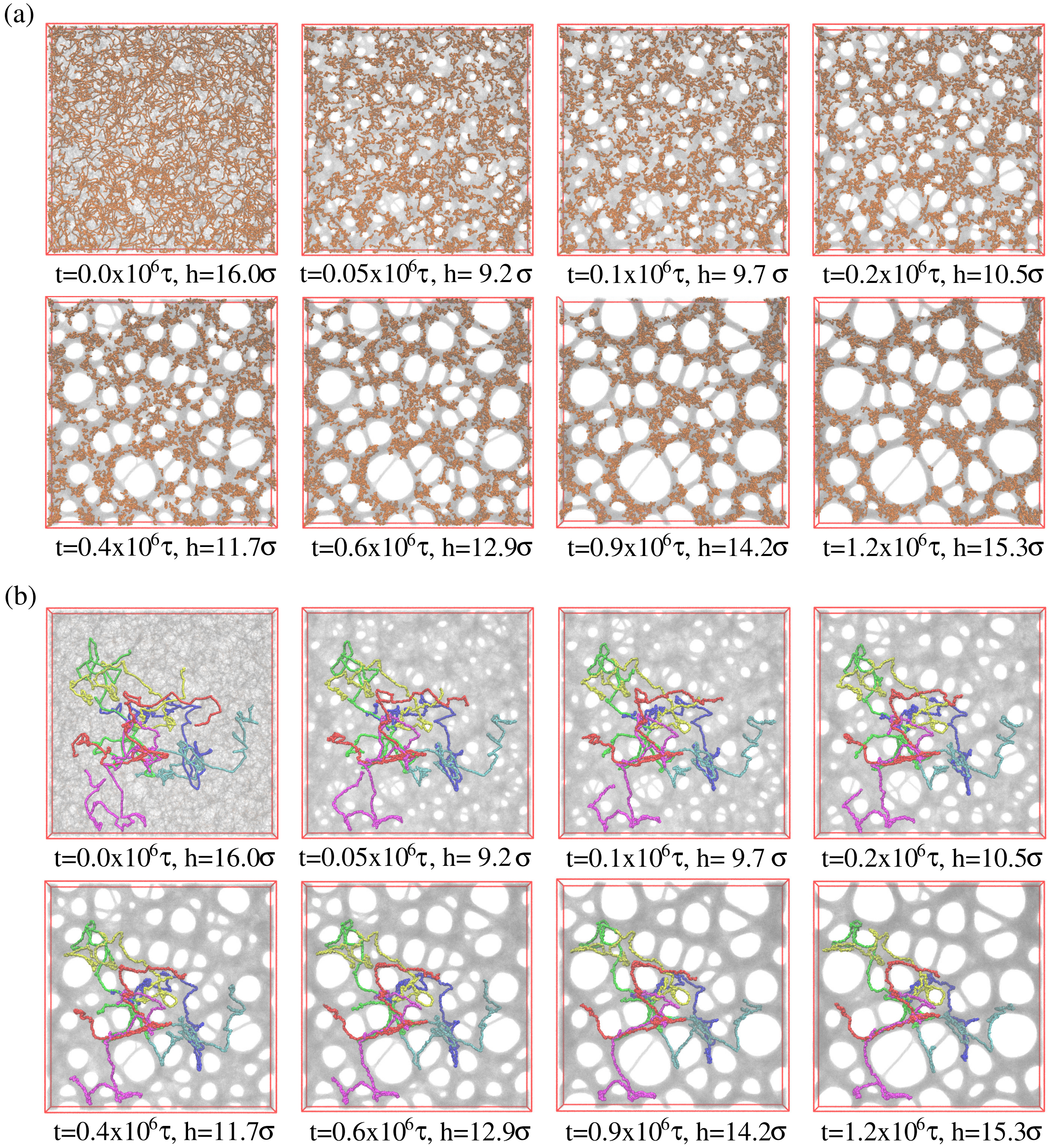


Figure S6. Snapshot configurations of thin polydisperse porous films at $\lambda \approx 4.0$ subject to relaxation at several selected relaxation times $t$ and assumed thicknesses $h$, as indicated where all 1000 chains of $N_2 = 100$ are marked in orange color (a), and the very same six selected chains of $N_1 = 1900$ are marked in different colors (b).

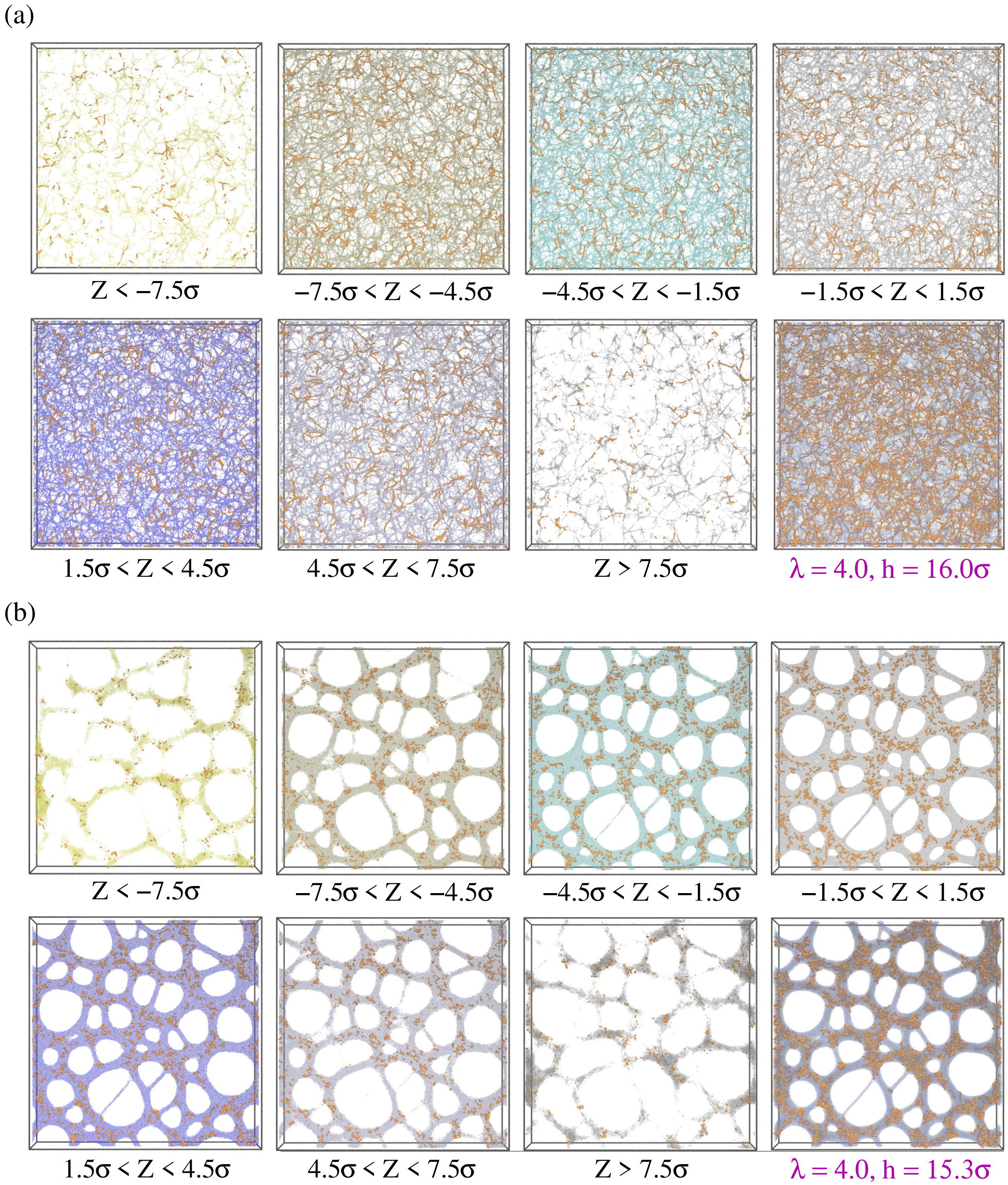


Figure S7. Snapshot configurations of slices of thickness $3.0\sigma$ cut along the lateral dimensions from the expanded film shown in Fig. S6 at $t = 0\tau$ (a) and $t = 1.2 \times 10^6\tau$ (b). Subchains belonging to long chains are shown in the background while belonging to short chains are marked in orange color with thicker bonds. The center of films is fixed at $z = 0\sigma$

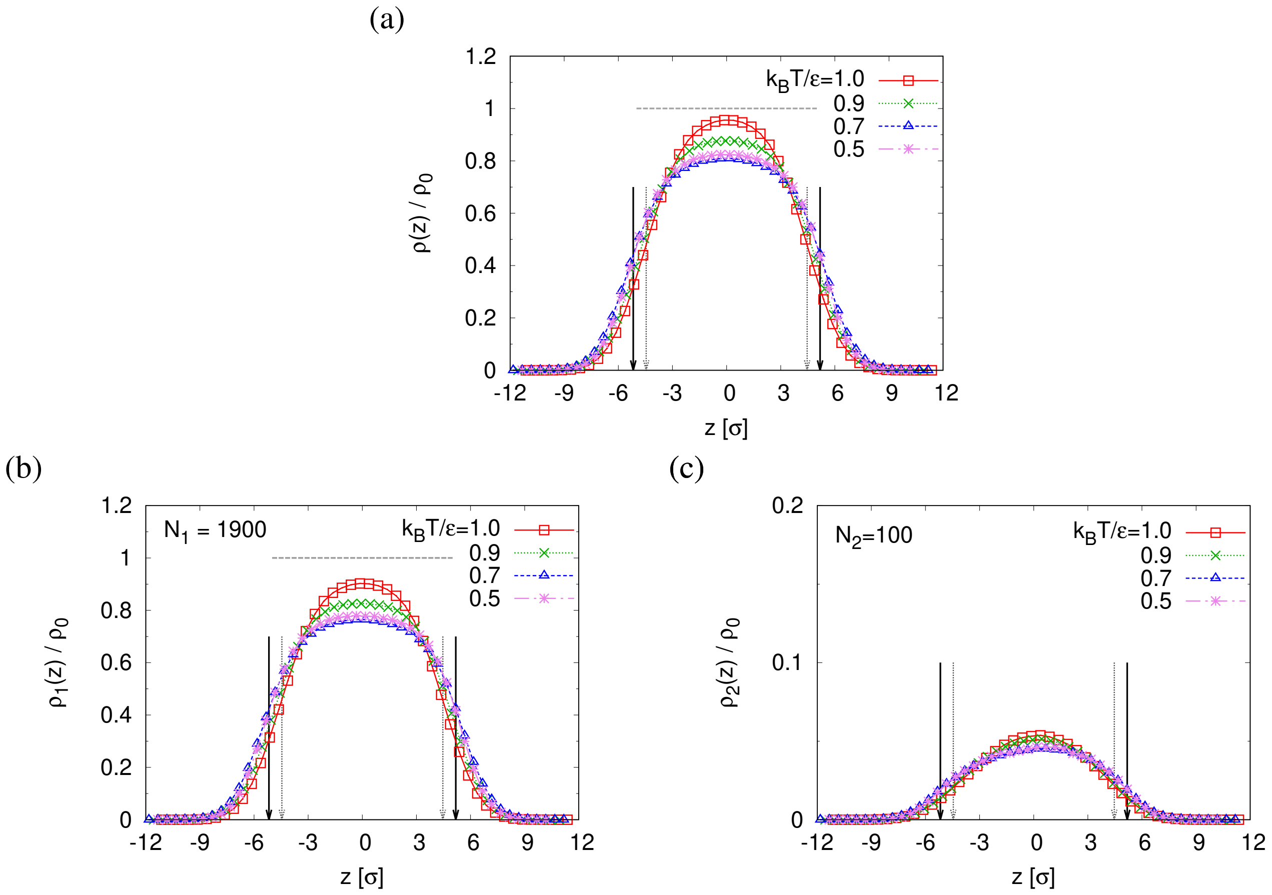


Figure S8. Rescaled monomer density profiles for all monomers, $\rho(z)/\rho_0$ (a), monomers in chains of length $N_1 = 1900$, $\rho_1(z)/\rho_0$ (b), and $N_2 = 100$, $\rho_2(z)/\rho_0$ (c), plotted as a function of $z$ at several selected temperatures $T$, as indicated. The centers of thin porous films in the $z$-direction are matched at $z = 0\sigma$. The interfaces located at $Z_G^{(\text{lower})}$ and $Z_G^{(\text{upper})}$ determined from $\rho(z)$ in (a) for films $k_BT/\epsilon = 1.0$ and 0.5 are indicated by dashed and sold arrows, respectively.
